# Reviving health mediation during the COVID-19 crisis and beyond: an implementation study in deprived neighbourhoods of Marseille, France

**DOI:** 10.3389/fpubh.2024.1313575

**Published:** 2024-07-03

**Authors:** Alix Fruleux, Jean Gaudart, Florian Franke, Steve Nauleau, Anne Dutrey Kaiser, Eva Legendre, Dorothée Balma, Marc Lescaudron, Lucile Tamalet, Philippe Malfait, Pascal Chaud, Stanislas Rebaudet

**Affiliations:** ^1^Ville de Marseille, Direction de la Santé publique et de l'Inclusion, Marseille, France; ^2^Aix-Marseille Université, Inserm, IRD, UMR1252 SESSTIM, ISSPAM, Marseille, France; ^3^Santé publique France, Saint-Maurice, France; ^4^Santé publique France, Marseille, France; ^5^Agence régionale de santé Provence-Alpes-Côte d'Azur (ARS Paca), Marseille, France; ^6^Prospective & Coopération, Marseille, France; ^7^Hôpital Européen, Marseille, France

**Keywords:** health mediation, community outreach, COVID-19, health education and awareness, testing, vaccination, disease hotspot, implementation science

## Abstract

**Introduction:**

In 2020, during France’s COVID-19 response, healthcare professionals from a hospital and an association initiated health mediation interventions in Marseille’s vulnerable neighbourhoods, funded by the regional health authorities. This mixed method research evaluates the CORHESAN program that lasted until June 2022.

**Methods:**

We examined CORHESAN documents and reports, conducted interviews, and analysed activity data, comparing it to the COVID-19 hotspots identified on a weekly basis at the neighbourhood level, using generalised linear mixed models (GLMMs).

**Results:**

CORHESAN was implemented by a team of up to nine health mediators, six private nurses hired on an *ad hoc* basis, supervised by a general coordinator and two part-time medical and nursing coordinators. Multiple partnerships were established with shelters, associations, social-housing landlords and local institutions. The team accompanied 6,253 people affected by COVID-19 or contact in the practical implementation of their isolation and contact tracing. Of the 5,180 nasopharyngeal samples for RT-PCR and 1,875 for antigenic testing: 12% were taken at home and 27% in partner facilities in the targeted neighbourhoods; 32% were taken from symptomatic patients and 30% in the context of contact tracing; and 40% were positive. Multiple awareness sessions on prevention methods and distributions of personal protection kits and self-diagnostic tests were conducted in the streets, in shelters, in associations or at home. A total of 5,929 doses of COVID-19 vaccine were administered in a walk-in vaccination centre, at temporary street vaccination posts, during operations at partner facilities, or during home-visits to patients with limited autonomy. GLMMs showed that the intervention significantly targeted its testing interventions in neighbourhoods with socioeconomic disadvantage and/or past under-testing (adjusted odds ratio (aOR), 2.75 [1.50–5.00]) and those with high hotspot level (aOR for level-3 versus level-0, 1.83 [1.24–2.71]).

**Discussion:**

The pandemic emphasised the potential of health mediation interventions to address health disparities. Building on this, a new program began in July 2022, aiming at enhancing cancer screening and vaccinations in deprived areas of Marseille. Evaluations are ongoing to assess its activities and impact, and provide evidence to future implementation initiatives.

## Introduction

The COVID-19 epidemic severely affected France and resulted in both a health and social crisis. The initial objective in managing the public health crisis in France was to reorganise hospitals in order to mitigate the death toll and ultimately slow down the transmission of the virus through a brutal but unavoidable non-pharmaceutical intervention: national lockdown (1–3). Gradually, the control of the epidemic also involved the promotion of barrier measures, the generalisation of screening tests and the implementation of targeted isolation, and was structured within the test-trace-isolate and then test-alert-protect (TAP) strategies, which aimed to massively test populations, alert contact cases and protect the others by establishing isolation to break transmission chains (4, 5). The crisis exacerbated social and territorial health inequalities among already vulnerable populations. It also highlighted the difficulty of implementing national recommendations for certain vulnerable populations: for example, home isolation was complicated by poor and overcrowded housing conditions (6–12).

The city of Marseille, located in the south-eastern region of Provence-Alpes-Côte d’Azur (PACA), remained distant from the epicentre of the first wave of COVID-19 in France between February and June 2020, thanks to the national lockdown (3). However, the city was heavily affected by the subsequent waves. Marseille is also characterised by a high population density and poverty, particularly in the centre and northern districts (11, 13, 14). As soon as 2020, several medical-social outreach initiatives emerged to provide support for suspected cases or contacts in SARS-CoV-2 screening and isolation. Notable examples included Nord Covid in the northern neighbourhoods of Marseille (with the Santé Environnement Pour Tous – SEPT association and the Médecins Santé Frontières – MSF non-governmental organisation), or Covid Homeless (15–17).

CORHESAN (for CORonavirus Hôpital Européen SANté) was conceptualised from April 2020 by practitioners and researchers in public health and infectiology from the SESSTIM research unit (Aix-Marseille Univ, INSERM, IRD) and Hôpital Européen Marseille, a general private non-profit hospital located in the impoverished 3rd district of Marseille. It was based on the experience of controlling cholera epidemics, especially in Haiti, where mobile teams of hygiene promoters or community mobilisers, water sanitation and hygiene (WASH) technicians and nurses were sent to the families and neighbours of cholera cases to identify additional cases, investigate local risk factors, raise awareness and distribute prevention kits (18–20). Mobile teams have also been successfully implemented against polio or Ebola outbreaks (21, 22). CORHESAN had the same objective of breaking the chains of transmission at the local level by increasing the population’s compliance with preventive measures, which has been shown to be influenced by socio-cultural factors and health literacy levels (9, 23–25). It also benefited from the experience of two similar outreach interventions against the pandemic, COVISAN in Paris (26), or YANACOV in French Guiana. CORHESAN was eventually launched in October 2020 by the Hôpital Européen Marseille and the association Prospective et Coopération, thanks to a pilot funding from the Provence-Alpes-Côte d’Azur Regional Health Agency (ARS PACA, the French state agency responsible for implementing national health policy in the regions). A core team of young mediators recruited from various non-healthcare backgrounds, accompanied by private nurses, physicians or pharmacists, was deployed in socially deprived neighbourhoods of the 1st to 3rd districts of Marseille, to offer confirmed or suspected patients assistance with isolation and contact tracing, as well as medical and social support. From February 2021, CORHESAN integrated the new national initiative MédiLAC (médiateurs de lutte anti-covid or *anti-covid mediators*), which aimed to strengthen the national test-alert-protect (TAP) strategy, and extended its activities to the entire 1st to 8th districts. It organised outreach activities in public places, within partner organisations, or during door-to-door visits, to provide information on prevention methods and the latest government doctrine on isolation and testing. The team also increased its SARS-CoV-2 testing services by RT-PCR or antigenic tests and distributed many self-tests. To better target these activities, the ARS communicated to CORHESAN and other MédiLAC of PACA region a weekly mapping of SARS-CoV-2 infection hotspots at the scale of neighbourhoods. From April 2021 onwards, CORHESAN also proposed outreach COVID-19 vaccination to people far from the healthcare system or unable to reach vaccination centres, in social housing facilities, at temporary points on public streets, directly at the homes of people referred by health professionals or met during door-to-door visits, and at a free walk-in centre located at the foot of the Hôpital Européen (see Table 1 for a description of the CORHESAN intervention model). These various activities continued until the end of the COVID crisis in June 2022. They successfully paved the way to the implementation by CORHESAN of a current outreach project, which aims to promote cancer screening and general vaccination uptake among the same deprived populations, and is based on health mediation, officially defined in France as a temporary process of “going outside the walls” and “going towards” populations, health and social professionals and institutions, as well as “working with” people in order to facilitate access to rights, prevention and care, and to make health professionals’ aware of access difficulties (27, 28).

**Table 1:** Description of the CORHESAN intervention model.

Main activities	Training and preparedness	Places of intervention in socially deprived neighbourhoods	Targeted population	Human resources and partners	Expected effects
1.Accompanying people affected by COVID-19 or contacts in isolation, contact tracing and offer medical and social support	Emphasis on empathy, confidentiality, respect of individual autonomy Training sessions based on field identified needs (i.e., family violence)	Hôpital Européen Patients’ home	Deprived population living in overcrowded housing People with limited autonomy	Health mediation team: Health mediator Nurse Physician Pharmacist Social worker Coordinator Community volunteer Other partners: Shelters Local associations Social-housing landlords Transit centres for Ukrainian refugees Institutional and academic partners: Hôpital Européen Regional Health Agency (ARS) National Public Health Agency (SpF) Public health researchers (SESSTIM)	Breaking the chains of transmission at the local level Enabling necessary conditions for containment Mitigate social and health impact of containment measures
2.Awareness sessions on prevention methods, latest policy on isolation, testing with distribution of personal protection kits and self-diagnostic test	Official initial then continuous training on COVID-19 policy response Training on COVID-19 medical management and orientation Training in health animation methods	Public streets Shelters Association At home during door-to-door visit	People with socioeconomic disadvantage and low health literacy		Increasing population’s compliance with preventive measure
3.Nasopharyngeal samples of RT-PCR and antigenic testing	Official initial training to perform diagnostic tests Weekly COVID-19 hotspot mapping Social deprivation and under-testing neighbourhoods mapping	CORHESAN hospital facilities At home during door-to-door visit Partner facilities Public streets Targeted neighbourhoods	Symptomatic patient Contact person People with limited autonomy		Improving testing rates in socially deprived and hotspot areas
4.COVID-19 vaccination	Training in general vaccinology and on COVID-19 vaccination Training in motivational interviewing applied to vaccination	Hôpital Européen free walk-in vaccination centre Temporary street vaccination posts During home visit Social housing facilities	People far from the healthcare system or unable to reach vaccination centres People referred by health professionals		Promoting access to vaccination and increasing vaccine coverage

To draw lessons from this innovative public health intervention in a time of crisis, the aim of this mixed methods study was to describe and analyse the implementation of the CORHESAN outreach project against COVID-19 in Marseille from October 2020 to June 2022.

## Methodology

### Study design and setting

The implementation of the CORHESAN intervention in Marseille during the period of November 2020 to June 2022 in response to the COVID-19 pandemic was described and analysed through a mixed methods study. The study combined a qualitative description of the intervention organisation, a narrative and quantitative report of implemented activities, and a quantitative ecological study of the targeting of SARS-CoV-2 outreach testing interventions towards weekly incidence hotspots.

With over 900,000 inhabitants, Marseille is the second-largest city in France. It is subdivided into 16 arrondissements or districts and 393 IRIS (the highest spatial resolution available for aggregated epidemiological data in France, known as “regrouped islets for statistical information”). These IRIS correspond to contiguous geographical areas grouping between 1,000 and 5,000 inhabitants. Marseille is densely populated and exhibits significant income disparities between neighbourhoods located in the northern (13th to 16th districts) and central (1st to 5th districts) parts of the city, and the neighbourhoods in the south (6th to 9th districts) and east (10th to 12th districts) (11, 13, 14). Nearly a quarter of the housing stock is over-occupied in the northern and centre neighbourhoods. Median incomes are also lower there than in the rest of the city.

### Data collection

#### Organisation and activities of CORHESAN

To describe the organisation of CORHESAN, we analysed the programmatic documents and progress reports of the project. From April 2022 to July 2022, AF also carried out participant observation within the team and conducted twenty semi-structured interviews with the coordination team, health mediators, correspondents at the regional health authorities, and key partners. The inductive analysis of interview themes highlighted the project’s history, organisation, strengths, weaknesses and perspectives. Quantitative data on the project’s activities were collected from the reporting tools set up by the team to monitor the project’s activities. They included the date, location and type of outreach interventions: isolation, contact-tracing and medical-social support; awareness on COVID-19 prevention measures; SARS-CoV-2 testing; and COVID-19 vaccinations. This activity data was anonymized and geographically aggregated at the district and IRIS levels, then compiled on a weekly basis.

#### COVID-19 hotspots

**Figure 1:**
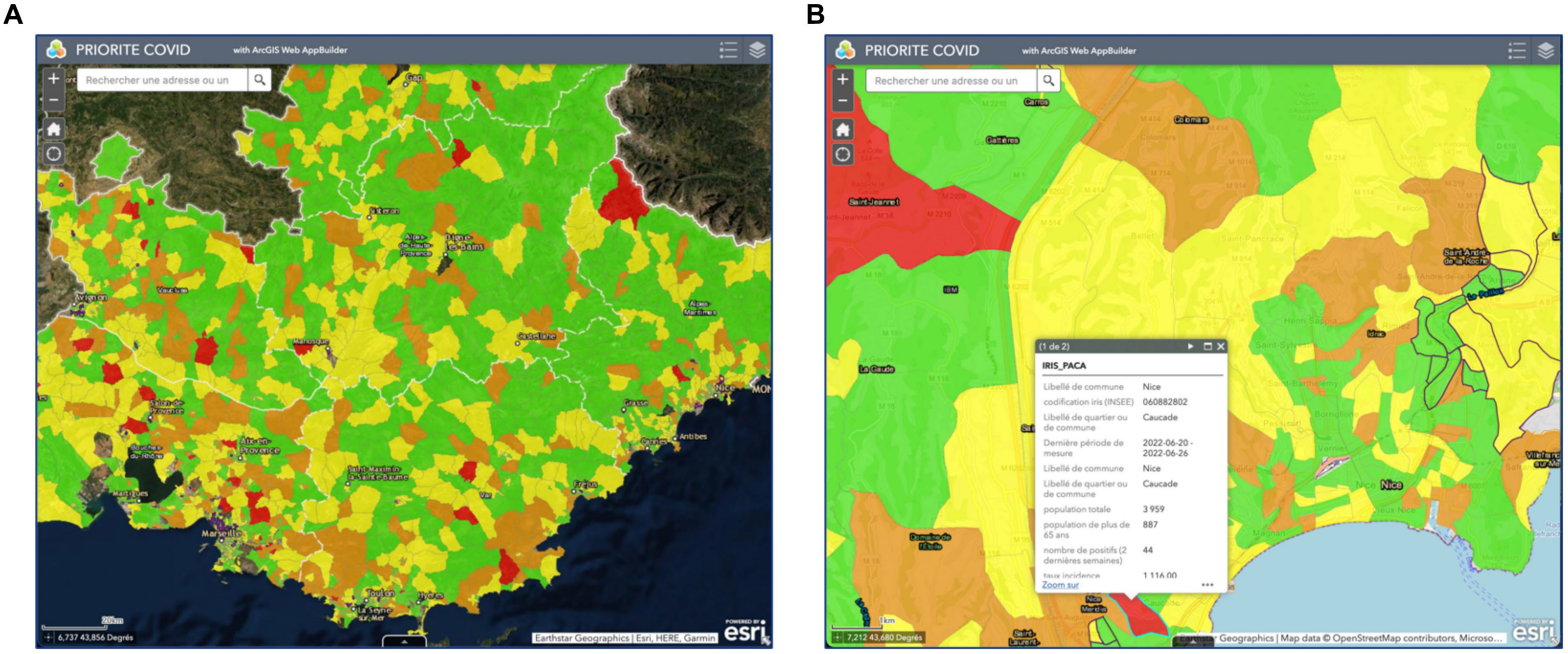
Screenshots of the weekly COVID-19 hotspot mapping **(A)** in the whole PACA region and **(B)** details provided after zooming and selecting a specific hotspot. The weekly hotspot identification combined analysis of the incidence rate of positive tests, the test positivity rate and the dynamics of the incidence rate at the IRIS level (data source: SI-DEP system).

To assess whether CORHESAN organised SARS-CoV-2 testing in the most relevant neighbourhoods of Marseille, we took advantage of a COVID-19 hotspot mapping project carried out from March 2021 to April 2022 by epidemiologists from the ARS (SN, CM), the regional unit of Santé publique France (FF, PC, PM) and an academic geo-epidemiology research group (JG, SR). Inspired by a cholera alert system implemented in Haiti (18), this analysis computed geocoded data from the SI-DEP system, a secure national platform that systematically recorded results of Covid-19 screening tests, and produced a weekly mapping of SARS-CoV-2 infection hotspots at the IRIS scale for the whole PACA region (Figure 1) (29, 30). The hotspot identification was based on the incidence rate of positive tests (+1 point if >80th percentile), the test positivity rate (+1 > 80th percentile) and the acceleration of the incidence rate (+1 > 80th percentile) over the past 15 days, which combined score (from 0 to 3) defined four hotspot levels: no hotspot, level 1, level 2 and level 3. Hotspot identification was complemented by the identification of IRIS areas with socio-economic deprivation and/or under-testing during the second wave of COVID-19 in 2020 (11). Briefly, 2017 general census data (including population age groups, population density, occupational categories, percentage of immigrants and foreigners) were analysed using principal component analysis followed by hierarchical clustering to define IRIS profiles describing local socio-demographic characteristics. The distribution of the social deprivation index FDep99 (31) confirmed the identification of a very deprived urban profile, and corresponding IRIS were tagged in the COVID-19 mapping. So were IRIS with SARS-CoV-2 testing rates below the departmental median rate and with a positivity rate > 10% in October 2020. The weekly analysis was mapped on an online ArcGIS Web AppBuilder (Figure 1). Each week, the list of IRIS hotspots and then the link to the online mapping was shared with the CORHESAN team and other MédiLAC teams to better target COVID-19 awareness on barrier gestures and testing outreach interventions in priority neighbourhoods. The complete list of COVID-19 hotspots from March 2021 to April 2022 in the 1st to 8th districts was obtained from the hotspot mapping team. Each IRIS-week (i.e., a particular IRIS during a particular week) was characterised as no hotspot, level 1, level 2 or level 3.

### Data analysis

#### Activity report

We first plotted the weekly evolution of the composition of the CORHESAN team and the number of outreach sessions by type and location. We then plotted the weekly evolution of SARS-CoV-2 tests performed by location, context and result, and the weekly evolution of COVID-19 vaccines administered by dose number and location.

#### Analysis of the CORHESAN SARS-CoV-2 testing interventions in response to COVID-19 hotspots

In a first analysis, response exhaustiveness was defined as the proportion of each hotspot that was targeted by at least one SARS-CoV-2 screening test (RT-PCR or antigenic test) in the same IRIS during the hotspot week or the following 2 weeks. To illustrate response exhaustiveness, we plotted and mapped the numbers of responded and non-responded hotspots per week and per IRIS, respectively. We then assessed the effect of several covariates on response exhaustiveness for each IRIS-week: hotspot level (3, 2, 1 or no hotspot); arrondissement or district of the IRIS; year; weekly number of active CORHESAN health mediators; distance from the CORHESAN headquarters at the Hôpital Européen. We used generalised linear mixed models (GLMMs) with hotspot response (responded vs. non-responded hotspot) as the independent variable and a binomial distribution (logistic model) (32). For the univariate analyses of IRIS and districts, each covariate was modelled separately as a unique random effect (Equation 1). For the univariate analyses of other covariates, we systematically included IRIS nested within districts as a common random effect in models where each covariate was modelled as a unique fixed effect variable (Equation 2). For the multivariate analysis, we included the fixed effect variables for which *p*-values were less than 0.25 (33), and IRIS nested within districts as a common random effect (Equation 3). The models estimated the crude odds ratio (cOR) and adjusted odds ratio (aOR) of response to alerts, and 95% confidence intervals (95%-CI) associated with each covariate.


(1) $$\mathrm{Logit}\left(\mathrm{Hotspot}:\mathrm{response}\right)=\left(1\;|\;\mathrm{IRIS}\right)∼\mathrm{Bin}$$

(2) $$\begin{array}{l} \mathrm{Logit}\left(\mathrm{Hotspot}:\mathrm{response}\right)=\mathrm{Hotspot}:\mathrm{level}+\\ \;\left(1\;|\;\mathrm{District}/\mathrm{IRIS}\right)∼\mathrm{Bin} \end{array}$$

(3) $$\begin{array}{l} \mathrm{Logit}\left(\mathrm{Hotspot}\_\mathrm{response}\right)=\mathrm{Hotspot}\_\mathrm{level}+\mathrm{Sociodisadv}\_\\ \mathrm{under}-\mathrm{testing}+\mathrm{Distance}\_\\ \mathrm{from}\_\mathrm{HE}+\mathrm{Mediators}\_\mathrm{No}+\\ \left(1\;|\;\mathrm{District}/\mathrm{IRIS}\right)∼\mathrm{Bin} \end{array}$$

#### Software

Data management was performed using Microsoft Excel for Mac v16.76. QGIS v3.22 (34) was used to calculate distance matrices and draw the maps. Graph design was performed using Microsoft Excel and Inkscape 1.1. R Studio version 2023.06.1 + 524 for Mac (35) with R version 4.3.1 for Mac and the {lme4} package (36) were used for statistical analyses.

#### Interviews

The semi-structured interviews conducted with the CORHESAN coordination team, health mediators, correspondents at the regional health authorities, and key partners were thematically analysed based on both notes and transcriptions. These qualitative results were integrated in the description of the team organisation and in the discussion section.

## Results

### CORHESAN team organisation

**Figure 2:**
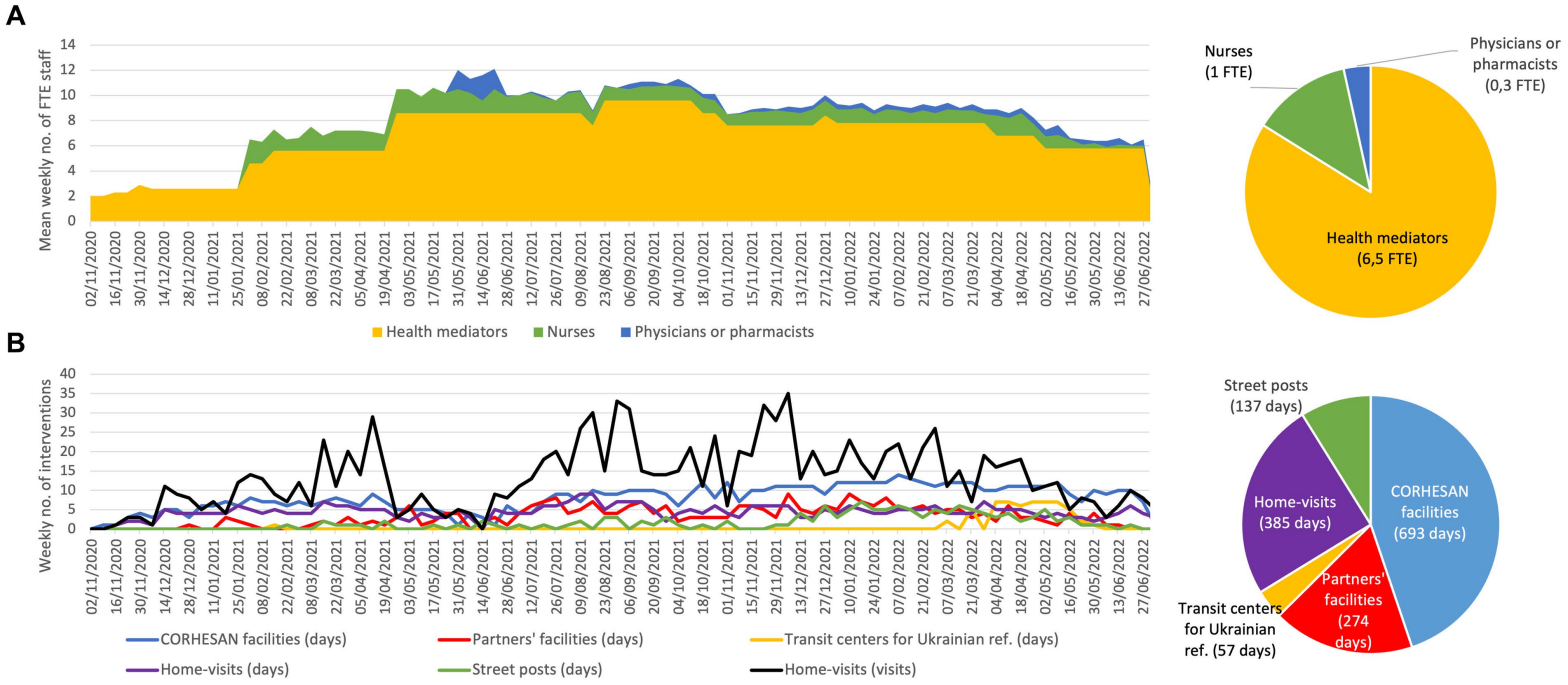
**(A)** Weekly evolution and mean number of full-time equivalent (FTE) health mediators, nurses, physicians and pharmacists participating in the outreach activities. **(B)** Weekly evolution and accumulated number of outreach sessions by type of location. Home-visits were summarised by weekly number of visits and by the number of days per week when visits were conducted. Ref., refugees.

To carry out its activities, the CORHESAN team consisted of a group of two to almost ten full-time equivalent (FTE) health mediators, and a pool of about 1 FTE private nurses and 0.3 FTE doctors or pharmacists who participated in activities on an *ad hoc* basis (Figure 2A). Mediators were recruited without any minimum qualification and none of them reported any previous experience in health mediation. Given the universality of COVID-19, they were not recruited specifically in the intervention neighbourhoods. All were open-minded, had excellent communication capacities and spoke several languages (including French, Arabic, Kabyle and various Berber dialects, Comorian, English, Spanish and Italian), which facilitated the team’s ability to adapt to different audiences. Besides, the diversity of their backgrounds (including hospital porter without higher education, scientific mediator, political science, project management, law, engineering, events sector, social volunteering…) created a rich and dynamic team with complementary skills and knowledge, and sometimes led to better public engagement through identification. Finally, to limit risks associated with initial COVID-19 severity, their age ranged from 22 to 27 years old. CORHESAN also included a part-time social worker. The coordination team consisted of a general coordinator with experience in coordinating projects in humanitarian contexts, a part-time coordinating infectiologist and epidemiologist with experience in community-level epidemic control strategies, and a part-time coordinating nurse manager with experience in patient therapeutic education. Volunteers from the beneficiary communities also regularly participated in the intervention’s activities. Finally, the intervention benefited from logistical and administrative support from the Hôpital Européen, including facilitated access to diagnostic tests and vaccines. With the intensification and diversification of activities, the outreach team was progressively divided into three sub-teams: a patient team; an education and testing team; and a vaccination team.

All health mediators received regular training on COVID-19, the latest updates on the ongoing national isolation and contact tracing policy, medical management, and prevention and vaccination strategy. They also received a specific two-day training organised by health authorities to enable them to perform nasopharyngeal swabs and collect diagnostic saliva samples for RT-PCR and rapid diagnostic tests. In addition, they received training in health animation methods, health literacy, and motivational interviewing applied to vaccination, delivered by experts in each respective field (37). Internal thematic training sessions, based on the needs identified by the team, such as dealing with family violence, were also provided by Hôpital Européen staff. The importance of respecting medical confidentiality, empathy and respect for individual autonomy was emphasised on a daily basis. This training was of particular importance for the health mediators, who did not initially come from the medical-social field.

The CORHESAN team gradually built up a large number of partnerships: the ARS, the regional unit of the National Public Health agency (Santé publique France, SPF), the health insurance, the municipal health and security services, the departmental council; hospitals, professional health organisations, city doctors, nurses, or pharmacists; social landlords, shelters for vulnerable people, social centres or animation centres, and local neighbourhood associations from civil society.

During the interviews, the mediators highlighted the crucial role of the hospital environment and their collaboration with nurses in familiarising themselves with the healthcare context and current health guidelines. Each mediator emphasised the importance of adaptability, both in coping with the ongoing evolution of the COVID-19 crisis management strategy and in effectively communicating and collaborating with diverse audiences and professionals. The complementarity of the recruited individuals’ profiles and the team’s multilingualism facilitated effective collaboration and adaptation to various on-the-ground requirements, as confirmed by several mediators.

### CORHESAN weekly activities

#### Interventions

From 2 November 2020 to 30 June 2022, the CORHESAN team welcomed the public for 693 days in its facilities at the Hôpital Européen. It organised 274 sessions at partner sites (social shelters, community associations…), 57 sessions at transit centres for Ukrainian refugees, 137 sessions at street posts and a total of 1,159 home visits for COVID-19 testing or vaccination (Figure 2B). In total, the team reached an estimated 10,880 people (data not shown).

#### Isolation, contact-tracing, and medical-social support

Every day, the CORHESAN team gathered the list of new SARS-CoV-2 positive patients from Hôpital Européen and Hôpital Saint-Joseph. Health mediators either phoned or visited every COVID-19 confirmed patient and/or family, who was admitted to the emergency room or hospitalised in order to collect the needs of their household in terms of support for isolation or contact tracing. When possible, contacts were invited to come and be tested directly within CORHESAN facilities at Hôpital Européen. A phone number was also dedicated to health professional or community associations or even individuals when symptomatic or contact people needed SARS-CoV-2 testing or support at home or at the hospital. If needed, the team proposed a home visit to test contacts, and to advise them for the practical implementation of their quarantine. CORHESAN could also organise direct assistance like temporary rehousing solutions, delivery of groceries, food parcels, meals, diapers, or infant formula, opening of health insurance rights, nurse visits, regular phone monitoring, referral to community associations… Over the period, the CORHESAN team provided such direct assistance to over 200 individuals. Unfortunately, the team confirmed that many data about medical-social support were missing from databases, and a more precise report was thus not feasible.

#### Awareness raising on COVID-19 prevention measures

CORHESAN staff shared the latest updates on the ongoing national isolation and contact tracing policy, medical management, and prevention and vaccination strategies to thousands of people (exact count unknown) during their interaction with the population in community associations and centres (women, children or teenagers groups…), in social shelters, at food distribution spots, or social round-ups. The aim was to raise their awareness of barrier gestures and vaccination and other prevention measures against COVID-19. The team distributed numerous protection kits, which included prevention leaflets, hydroalcoholic gel and masks. At the request of the health authorities, they also provided 11,400 SARS-CoV2 unsupervised antigenic rapid diagnostic tests (self-tests). As illustrated by interviews, the team sometimes simplified some complex and ever-changing governmental contact-tracing and isolation protocols to better fit with the health literacy of the targeted population and facilitate the practical organisation of operations. This could include harmonising testing delays and isolation periods for family contacts, or not testing younger children within households where isolation was not possible. The mediators also regularly phoned back patients and contacts to remind them of the recommendations for isolation, prevention and retesting.

#### SARS-CoV-2 testing

**Figure 3:**
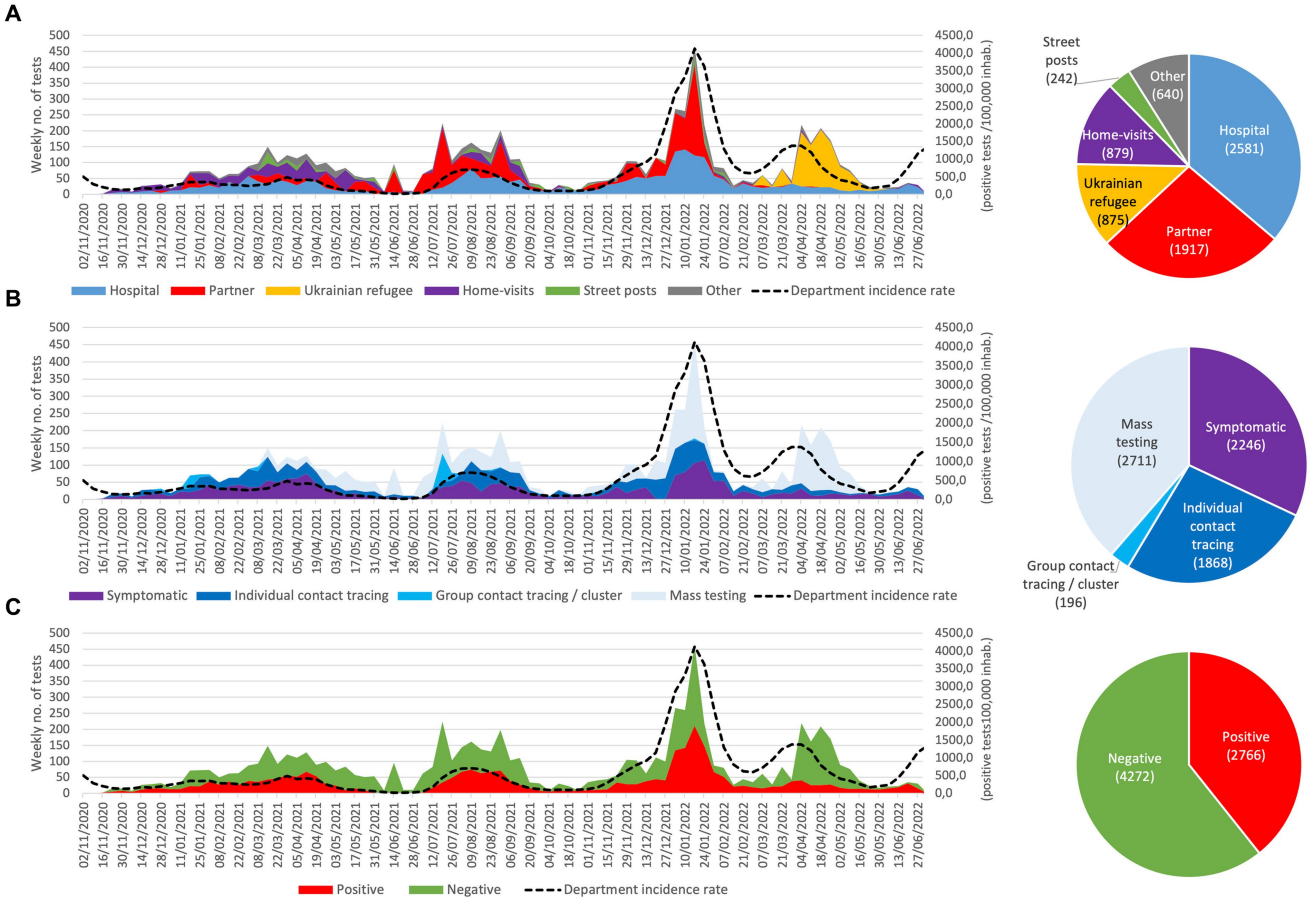
Weekly evolution and accumulated number of SARS-CoV-2 tests performed by the CORHESAN team, by **(A)** location, **(B)** context, and **(C)** results. (data source for case incidence rate in the Bouches-du-Rhône department were Marseille is located: https://www.data.gouv.fr/fr/datasets/donnees-de-laboratoires-pour-le-depistage-a-compter-du-18-05-2022-si-dep/).

On this period, CORHESAN performed 7,093 diagnostic tests for SARS-CoV-2 on 6,252 individuals, including 72% RT-PCR tests and 26% antigenic tests using nasopharyngeal swabs, as well as 1% saliva-based RT-PCR tests in children (data not shown). The tests were carried out in various locations: 36% within CORHESAN hospital facilities, 27% at partner sites (social shelters or community associations…), 12% within transit centres for Ukrainian refugees (from March 2022 on, before entering emergency accommodation in Marseille), 12% during home visits, and, as part of the MédiLAC missions assigned by ARS, 3% on public streets (Figure 3A). In total, 32% of the tests were sampled from individuals with suggestive symptoms of COVID-19, 27% as part of contact tracing efforts, 3% as part of community cluster investigations (at the request of health authorities when more than three positive cases were detected in collective housing structures or other communities), and 39% during mass screening activities on public places (Figure 3B). Out of all these tests, 2,507 (39%) were found to be positive (Figure 3C). The weekly number of performed tests followed the successive case incidence waves of the COVID-19 pandemic (Figure 3).

#### COVID-19 vaccinations

**Figure 4:**
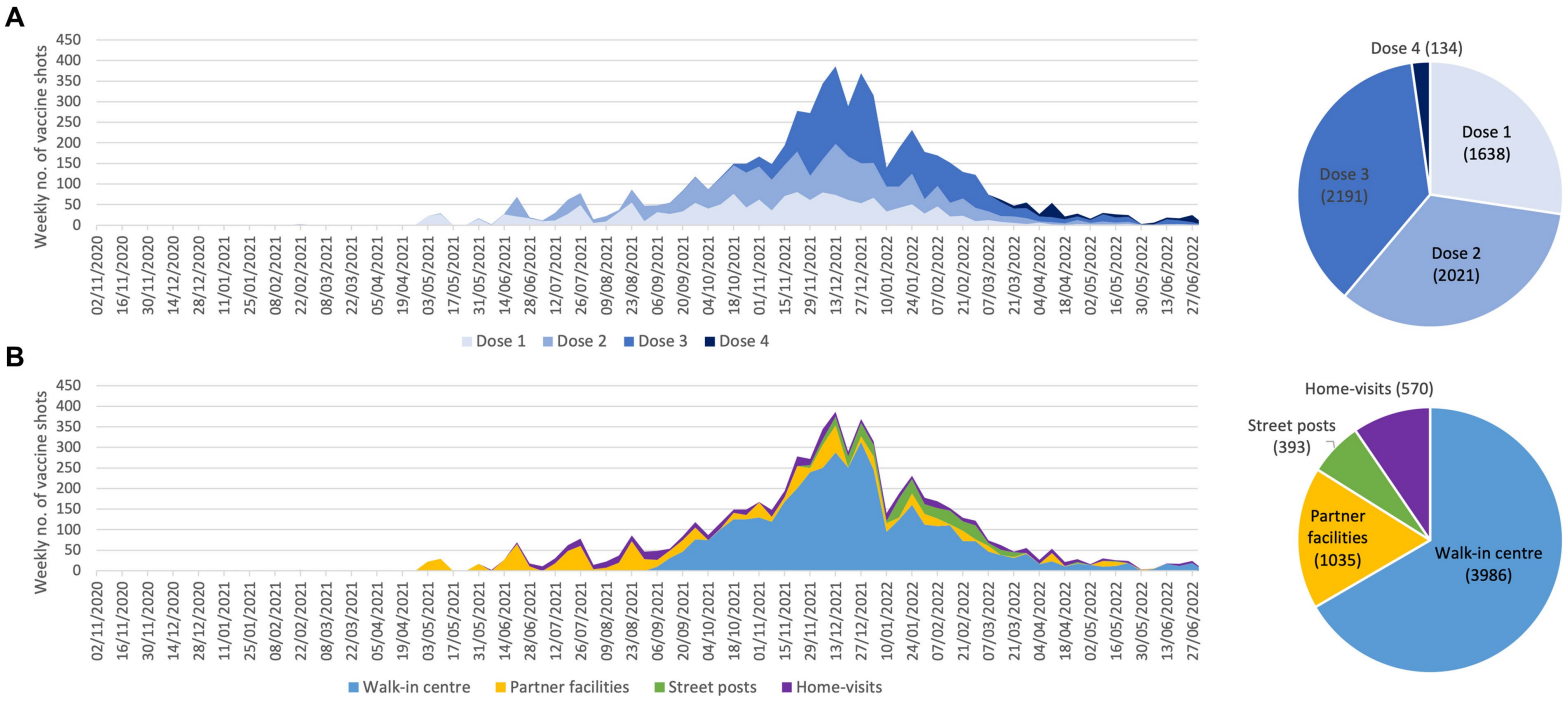
Weekly evolution and accumulated number of COVID-19 vaccine doses administered by the CORHESAN team, by **(A)** dose number and **(B)** location.

According to interviews of the CORHESAN coordination team, the project could build on the Hôpital Européen Marseille, which received COVID-19 vaccines in January 2021 as soon as they became available. After informing the ARS, the CORHESAN medical coordinator, as well as the vaccination centre coordinator and managing pharmacist of the Hôpital Européen took responsibility for organising outreach vaccination interventions, before it was included in the national prevention strategy. From January 1, 2021, to June 30, 2022, the CORHESAN team thus administered 5,984 doses of COMIRNATY^®^ (BioNTech-Pfizer) to 4,628 persons. The peak of vaccination occurred in November–December 2021 (Figure 4). Out of these 5,984 shots, 27% were first doses, 34% second doses, 37% third doses and 2% were fourth doses (Figure 4A). Most of them (67%) were administered in a free walk-in vaccination centre located at the foot of the Hôpital Européen, 17% at partners’ facilities, 7% on street vaccination posts, and 9% during home-visits (Figure 4B). Interestingly, 387 first doses could even be administered between January and June 2022, notably on street posts (13%), at partners’ (13%) (data not shown).

### Targeting of interventions

From November 2020 to January 2021, the CORHESAN team targeted the 1st, 2nd, and 3rd districts of Marseille. Starting from February 2022, at the request of the ARS and as part of the integration of CORHESAN into the national MediLAC strategy, the team expanded its actions to also include the 4th to 8th districts, while the 9th to 16th districts were covered by the SEPT association.

According to the different interviews, the weekly COVID-19 hotspot mapping, which computed geocoded test results and was provided by ARS and SPF, enabled the CORHESAN team to prioritise the awareness sessions or SARS-CoV-2 mass screening interventions in neighbourhoods where the virus was more actively circulating, or where screening was structurally deficient. Mediators also highlighted that familiarity with the city was a significant advantage in determining which hotspots were most relevant for intervention. Hotspots regularly led to actions in public places and door-to-door. In addition, the hotspots prompted numerous “exploratory” visits to neighbourhoods to identify new partners, particularly in the social and cultural sectors, with whom to organise awareness-raising events, and reach the most vulnerable populations (in the streets, hotels, squats, slums, etc.). Some interventions were also organised at the direct request of regular partners. Two of them particularly emphasised that the team’s good familiarity with current prevention protocols and the repetition of interventions provided an effective platform for confidently discussing COVID-19 with the target audience. The ARS also regularly involved CORHESAN in cluster management with testing and isolation advice. Several mediators highlighted the team’s good responsiveness and ability to reorganise for this cluster response. Finally, several team members mentioned the importance of adapting to the current context in order to target interventions, such as scheduling awareness activities at food distributions during Ramadan.

**Figure 5:**
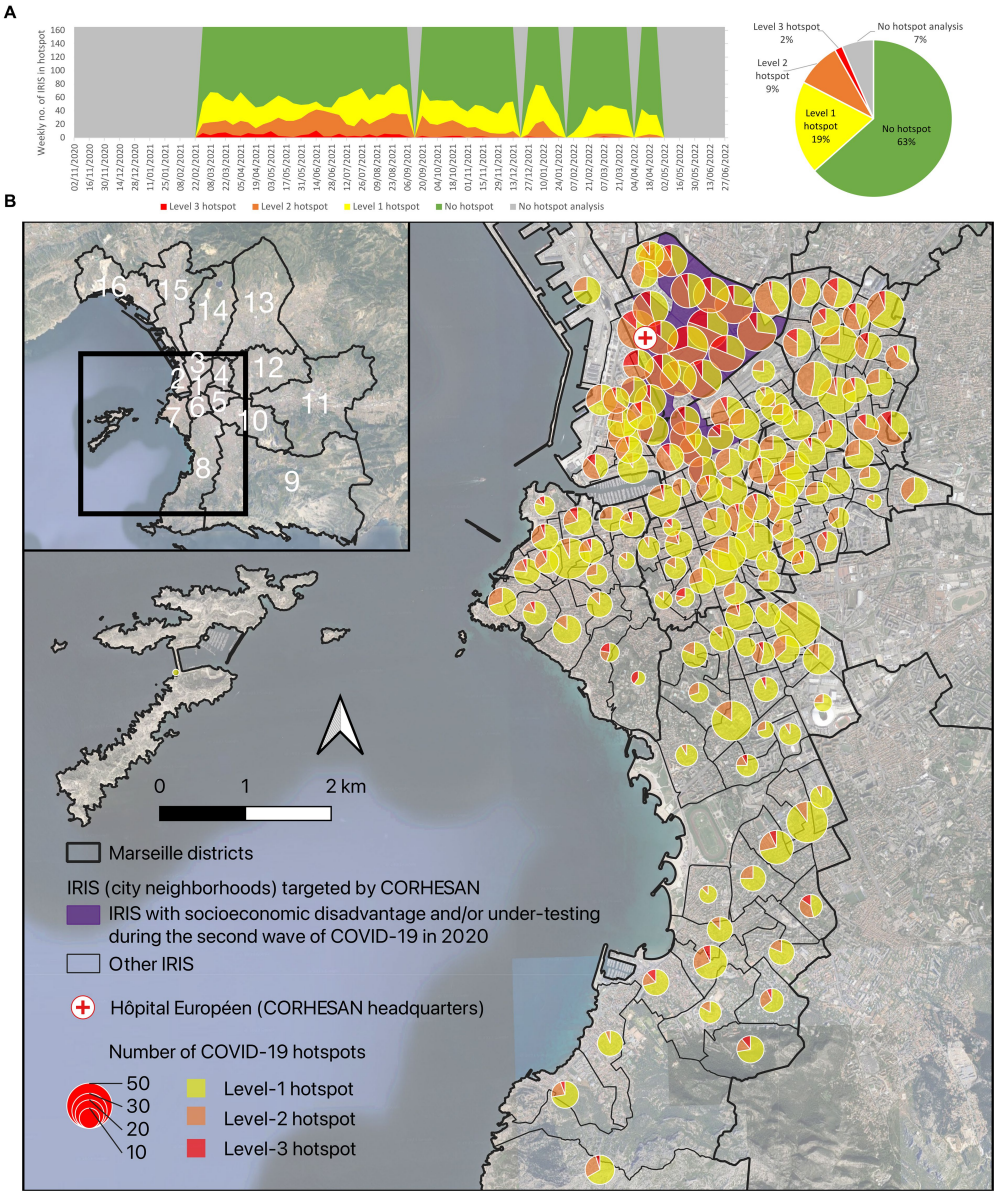
**(A)** Weekly evolution and **(B)** geographical distribution of the different COVID-19 hotspot levels, between March 2021 and April 2022, in the 165 IRIS covered by the CORHESAN team. The weekly hotspot identification combined analysis of the incidence rate of positive tests, the test positivity rate and the dynamics of the incidence rate at the IRIS level (data source: SI-DEP system).

To quantitatively assess the targeting of CORHESAN’s SARS-CoV-2 testing interventions, we retrospectively compared them with COVID-19 hotspots. During the 61 weeks from March 2021 to April 2022, the COVID-19 hotspot mapping group performed 57 weekly analyses (covering 93% of the period), with four missing reports in September 2021, during the 2021 Christmas period, in January and in April 2022 (Figure 5A). Among the 165 IRIS covered by CORHESAN over this 61-week period, level-3 hotspots concerned 159 (2%) of the 9,405 IRIS-weeks, occurred almost exclusively in 2021 (Figure 5A), and mostly clustered in the 3rd (42%), 4th (15%), 8th (11%) and 2nd (10%) districts (Figure 5B). Level-2 hotspots concerned 10% of IRIS-weeks (12% in 2021 and 3% in 2022) (Figure 5A), and mostly located in the 3rd (31%), 4th (15%), 1st (13%), 8th (11%) and 2nd (10%) districts (Figure 5B). Finally, level-1 hotspots concerned 21% of IRIS-weeks and mostly clustered in the 8th (24%) district (Figure 5B). A total of 44% of the 159 level-3 hotspots located in IRIS with socioeconomic disadvantage and/or under-testing, 34% of the 927 level-2 and 12% of the 1,933 level-1 hotspots (Figure 5B).

**Figure 6:**
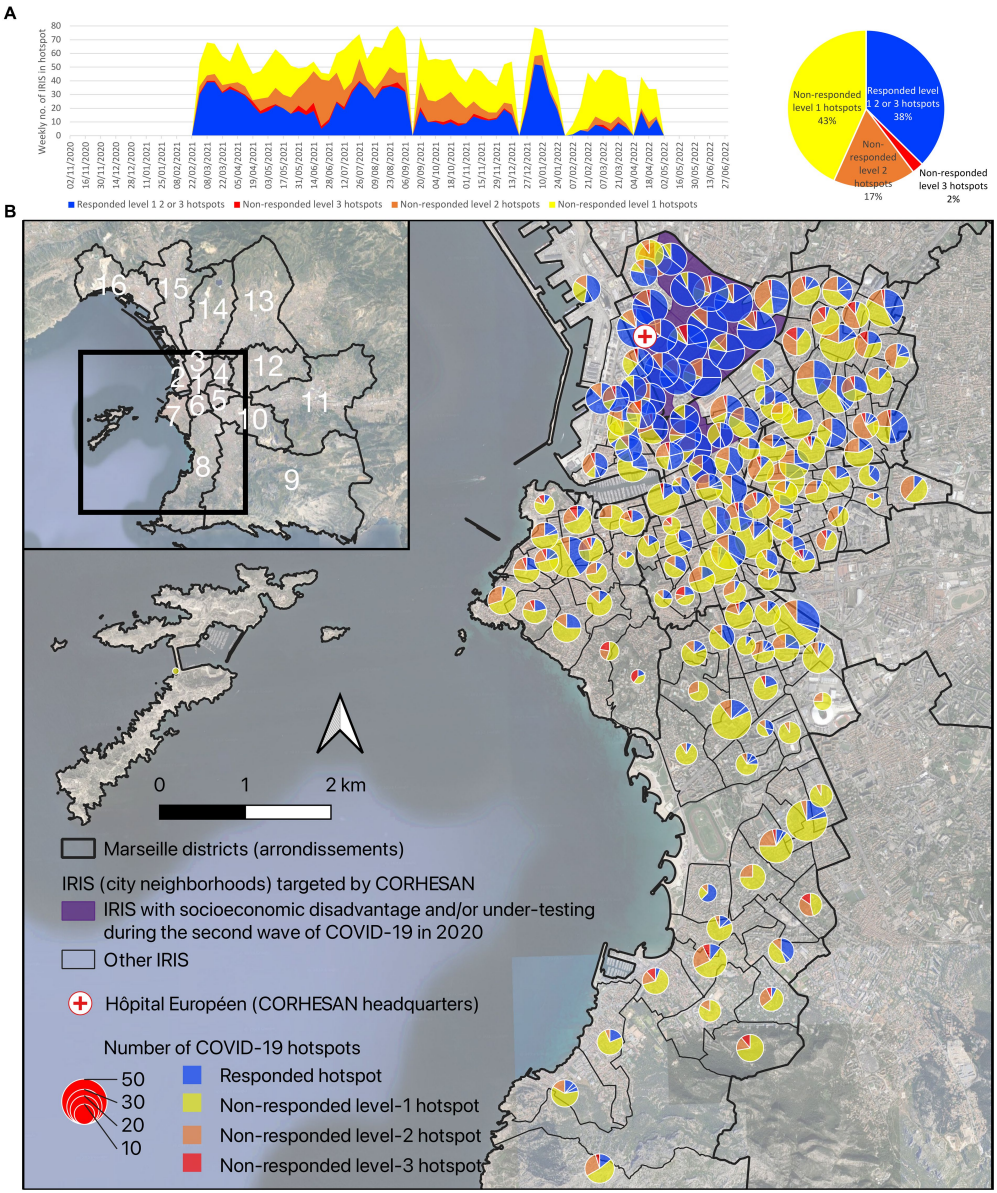
**(A)** Weekly evolution and **(B)** geographical distribution of responded and non-responded level-1 to level-3 COVID-19 hotspots, between March 2021 and April 2022, in the 165 IRIS covered by the CORHESAN team. Hotspots were considered as responded if the CORHESAN team performed at least one SARS-CoV-2 screening test (RT-PCR ou antigenic test) in the same IRIS during the hotspot week or the two following weeks.

To assess whether CORHESAN had targeted these hotspots, we considered an hotspot as responded if the team performed at least one SARS-CoV-2 screening test (RT-PCR or antigenic test) in the same IRIS during the hotspot week or the two following weeks. Overall, 1,129 (37%) of the 3,019 hotspots were responded, and the specific response proportion was 32, 45 and 55% for level-1 -2 and − 3 hotspots, respectively (Table 2; Figure 6A). The proportion of responded hotspots was nearly similar in 2021 (38%) and 2022 (35%) (Table 2; Figure 6A). It was much higher in the 3rd (73%), 2nd (57%) and 1st (48%) than in the other districts, including only 18 and 17% in the 7th and 8th southern districts of Marseille (Table 2; Figure 6B). The proportion of responded hotspots was 74% in IRIS with socioeconomic disadvantage and/or under-testing, and 28% in other IRIS (Table 2; Figure 6B). As a matter of fact, IRIS with socioeconomic disadvantage and/or under-testing clustered in the 3rd, 2nd and 1st districts (Figure 6B). The mean distance from Hôpital Européen, headquarters of the CORHESAN team, to responded hotspots was 1.9 km (SD, 1.5), whereas it was 3.3 km (2.0) to non-responded hotpots (Table 2; Figure 6B). Finally, the mean number of active health mediators in the CORHESAN team was 7.7 (SD, 1.3) during the week of responded hotspots, and 8.1 (1.1) during the week of non-responded hotspots.

**Table 2:** Exhaustiveness of response to COVID-19 weekly hotspots between March 2021 and April 2022, in the 165 IRIS covered by the CORHESAN team: factors associated with the odds of response (logistic mixed models).

		IRIS-weeks			Univariate analysis^b^^c^		Multivariate analysis^d^	
		Total	Responded	Non-responded	cOR [95%-CI]	*p*-value	aOR [95%-CI]	*p*-value
Number of IRIS-week (%)		9,405	2,704 (29%)	6,701 (71%)				
Number of IRIS-week with a COVID-19 hotspot (%)		3,019 (32%)	1,129 (37%)	1890 (63%)				
IRIS						<0.0001^b^		
District or arrondissement^a^						<0.01^b^		
	1st	319 (11%)	154 (48%)	165 (52%)				
	2nd	274 (9%)	156 (57%)	118 (43%)				
	3rd	562 (19%)	412 (73%)	150 (27%)				
	4th	416 (14%)	121 (29%)	295 (71%)				
	5th	277 (9%)	56 (20%)	221 (80%)				
	6th	306 (10%)	81 (26%)	225 (74%)				
	7th	270 (9%)	49 (18%)	221 (82%)				
	8th	595 (20%)	100 (17%)	495 (83%)				
IRIS and district random effect						<0.01		<0.0001
Hotspot level, *n* (%)								
	No hotspot	6,386 (68%)	1,575 (25%)	4,811 (75%)	Ref	Ref	Ref	Ref
	Level 1	1933 (21%)	626 (32%)	1,307 (68%)	1.20 [1.05–1.37]	<0.01	1.21 [1.06–1.38]	<0.01
	Level 2	927 (10%)	416 (45%)	511 (55%)	1.20 [1.01–1.43]	0.03	1.21 [1.16–1.64]	<0.001
	Level 3	159 (2%)	87 (55%)	72 (45%)	1.83 [1.24–2.71]	<0.01	1.85 [1.24–2.77]	<0.01
Socioeconomic disadvantage and/or under-testing, *n* (%)^a^								
	No	2,389 (79%)	663 (28%)	1726 (72%)	Ref	Ref	Ref	Ref
	Yes	630 (21%)	466 (74%)	164 (26%)	2.67 [1.45–5.00]	<0.01	2.75 [1.50–5.00]	<0.0001
Distance from Hôpital Européen Marseille, mean (SD; km)^a^		2,7 (2)	1,9 (1,5)	3,3 (2)	0.77 [0.66–0.89]	<0.001	0.77 [0.68–0.88]	<0.0001
Year, number (%)^a^								
	2021	2,369 (78%)	902 (38%)	1,467 (62%)	Ref	Ref	ND	ND
	2022	650 (22%)	227 (35%)	423 (65%)	0.94 [0.83–1.06]	0.29	ND	ND
Number of health mediators, mean (SD)^a^		7,9 (1,2)	7,7 (1,3)	8,1 (1,1)	0.76 [0.72–0.79]	<0.0001	0.75 [0.73–0.78]	<0.0001

a Factors summarised for the 3,019 IRIS-weeks with a COVID-19 hotspots only, IRIS-weeks with no hotspots being excluded.

b For each of these univariate analyses, IRIS and districts was modelled as a unique random effect variable.

c For these univariate analyses, IRIS and districts were modelled as random effect variables, with IRIS nested within districts. Models provided a common *p*-value for both random effects.

d For the multivariate analysis, the model included IRIS and districts as random effect variables, with IRIS nested within districts, and all fixed variables for which univariate *p*-value was < 0.25 The model provided a common *p*-value for random effect variables.

Hotspots were considered as responded if the CORHESAN team performed at least one SARS-CoV-2 screening test (RT-PCR ou antigenic test) in the same IRIS during the hotspot week or the two following weeks.SD, standard deviation; cOR, crude odds ratio; aOR, adjusted odds ratio; 95%-CI, 95% confidence interval; ND, no data (variables not included in the multivariate analysis).

Using multivariate GLMMs, the odds of responding to a COVID-19 hotspots (exhaustiveness) appeared influenced by the IRIS, and the district (common *p*-value of random effects <0.05) (Table 2). Exhaustiveness of response to hotspots was higher for level-3 hotspots (adjusted odds ratio (aOR), 1.83 [1.24–2.71]; *p*-value <0.01) than for level-2 (aOR, 1.20 [1.01–1.43]; *p*-value <0.001), level-1 hotspots (aOR, 1.21 [1.06–1.38]; *p*-value <0.01) and IRIS with no hotspot (reference class). Exhaustiveness of response to hotspots was higher for hotspots affecting IRIS with socioeconomic disadvantage and/or under-testing (aOR, 2.75 [1.50–5.00]; *p*-value <0.0001). Exhaustiveness of response to hotspots decreased when the IRIS was far from CORHESAN headquarters (aOR, 0.77 [0.68–0.88] per km; *p*-value <0.0001). Finally, response was less likely during weeks with more active health mediators (aOR, 0.76 [0.72–0.79] per mediator; *p*-value <0.0001) (Table 2).

## Discussion

This mixed methods study provides a unique perspective on the implementation of a 19-month original outreach and health mediation intervention dedicated to COVID-19 mitigation for some deprived populations in the second largest city in France (see project’s main barriers and facilitators in Table 3). The study was made possible by the prospective collection of all intervention reports and activity data. However, CORHESAN was not designed as a research project, which led to imprecise data collection on support services, awareness sessions, distribution of protection kits, or the living IRIS of vaccinees.

**Table 3:** CORHESAN’s main barriers and facilitators.

	Main barriers	Main facilitators
Context	Constant adaptation required due to the ever-changing national doctrine on isolation and contact tracing Public mistrust of the COVID-19 vaccination, amplified by local influential figures such as Didier Raoult Lack of professional recognition of health mediation by the French authorities, sometimes leading to reduced institutional engagement Difficulty engaging outreach interventions with institutions like the National Education due to a culture of institutional compartmentalisation between health and education ministries Difficulty in deploying new outreach interventions such as street actions, due to administrative delays in obtaining the necessary authorisations	Lessons learned from past outreach interventions against other diseases such as cholera National MédiLAC program contributing to project legitimacy and team training Health crisis fostering significant integration between the health and social sector Health crisis fostering collaboration between academic researchers, state agencies and field actors Health crisis fostering local civil society initiatives and dynamic community engagement COVID-19 epidemiological data provided by the government to be analysed by local epidemiologists and shared with field actors to target interventions
Team composition	Lack of peer mediators from certain specific communities enabling easier access to them	Team’s multidisciplinary nature (health mediator, private nurses, physicians, pharmacist, social worker, coordinators, community volunteers) facilitating knowledge and skill exchange as well as innovation Team’s multilingualism and background diversity facilitating effective collaboration and adaptation to various contexts Teams’s adaptability enabling quick response to the evolving pandemic situation
Hospital anchorage (Hôpital Européen)		Effective administrative, technical and logistical support Good professional training of mediators through proximity to doctors, nurses, biologists, social workers, and through regular visits to hospitalised COVID-19 patients Facilitated access to medical and social advice and care for vulnerable patients encountered in the field Increased confidence of the target population and institutions thanks to the hospital’s reputation Secured and GDPR compliant storage of the project’s electronic health records on hospital servers Easy access to a wide range of medical supplies including personal protection equipment, swabs, rapid diagnostic tests, COVID-19 vaccines, first aid and resuscitation bags in case of vaccine side effects… Financial solidity that allowed the continuation of interventions during gaps between funding periods Administrative facilities for hiring nurses on an hoc basis Motivation of hospital staff to get involved in an innovative outreach project that was seen as useful
Dual association anchorage (Prospective et Coopération)	Increased complexity of the financing agreement between health authorities, Hôpital Européen and Prospective et Coopération	Administrative flexibility to hire project coordinators with NGO experience, compensate volunteers from the target communities
Tools used by the team	Difficulties for the CORHESAN team to collect the large amount of intervention data produced	Dedicated spreadsheets for routine collection of intervention activities for project reporting and scientific evaluation Weekly COVID-19 hotspot mapping helping CORHESAN team to target interventions COVID-19 hotspot mapping enabling to quantitatively evaluate the targeting of the SARS-CoV-2 testing interventions
Funding	Difficulty for the team to plan for the long term due to short-term contracts and project funding that were renewed incrementally in response to the constantly evolving pandemic situation	MédiLAC program helping secure funding between February 2021 and June 2022 Convenient and flexible financial support enabling innovative action and continuous project evolution Sustainability ensured through significant funding and ongoing team training

This intervention strategy, adapted from successful interventions against other diseases such as cholera, addressed not only the pandemic but also the exacerbated social and territorial health inequalities (8, 10–12). It aimed to provide practical support for isolation, quarantine and contact tracing, awareness of preventive measures, SARS-CoV-2 testing and COVID-19 vaccination to populations likely to need it most. Among the few outreach initiatives launched in France in 2020 (26), CORHESAN influenced government discussions and contributed to the establishment of the MédiLAC national strategy from February 2021. More generally, WHO and UNICEF published guidance on the use of community health care workers in the context of the COVID-19 pandemic in 2020 (38), and several other outreach experiences have been described abroad, to promote appropriate symptom management, preventive behaviours, testing or vaccination in French Guiana (39, 40), in the USA – particularly among the Latinx community (41–51), in Mexico (52), in the UK (53), in Kenya, Senegal and Uganda (54), in Bangladesh (55) or in Indonesia (56).

CORHESAN has been at the vanguard of the re-emergence of health mediation as a public health tool for health promotion. In France, health mediation is officially defined as a temporary process of “going outside the walls” and “going towards” populations, health and social professionals and institutions, as well as “working with” people, with openness, with respect and without judgement, in order to facilitate access to rights, prevention and care, and to make health professionals’ aware of access difficulties (27, 28). This corresponds to the CORHESAN team’s objectives and positioning. Third-party mediators have been called “health mediators” or “navigators” in France, “community health workers” in the English-speaking world, or “promotores” in Latin America, and they have addressed a wide range of interventions (28). Over the last 20 years in France, many health associations have relied on mediators to help individual patients navigate the healthcare system or to promote healthy behaviours (28, 57, 58). However, there has been little scientific evaluation of these experiences and no professional recognition of health mediation by the French authorities (28). Only two academic training programs have been available in the country (Paris and French Guiana) (28). In addition, mediation does not seem to have been used in the context of a health crisis in recent decades. During the COVID-19 pandemic, when health professionals were in short supply, CORHESAN – and other MédiLAC projects – were able to recruit specific health mediators, train them in many aspects of COVID-19 care and prevention, as well as in health animation methods, health literacy and motivational interviewing applied to vaccination. They could even habilitate them for SARS-CoV-2 testing, and use them to organise medical-social support at home, awareness and testing sessions, or vaccination campaigns.

CORHESAN assembled an unusual team of health mediators employed by a hospital, private nurses, hospital and private practitioners, and volunteers from the local communities. The team worked closely in a network with a wide range of local actors. Emphasis was placed on empathy and respect for individual autonomy, in particular through training in motivational interviewing, as a way of restoring public confidence in isolation, quarantine, contact tracing or COVID-19 vaccination in a context of restrictive and authoritarian policies (25, 59). It appeared all the more important in Marseille, where the popularity and influence of the local and highly controversial Didier Raoult seemed to be at its height. CORHESAN’s interventions also aimed to strengthen health democracy, although the strategy, implemented in the context of a health crisis to carry out interventions promoted by the health authorities, was not initiated by the community (60). They also benefited from being delivered by a local hospital, which increased the confidence of the target community and of the funding institutions. Extensive partnerships with health professionals, local institutions, community associations or social landlords reinforced the comprehensive approach of the team. The use of intervention management strategies drawn from humanitarian practice ensured effective coordination, innovation and responsiveness.

CORHESAN’s outreach interventions also benefited from another innovation, the COVID-19 hotspot mapping project (29, 30), which was inspired by a cholera alert system implemented by the Haitian health authorities from 2013 to 2019 (18), and seems to remain a unique initiative in France and worldwide for COVID-19. This mapping was made possible by the availability of SI-DEP, a national centralised COVID-19 test data. Hotspot analysis was carried out for the entire PACA region, including Marseille, on a weekly basis, and at the IRIS scale, the finest French administrative division, although geocoding and spatial aggregation were hampered by the sometimes poor quality of patient addresses notified by the medical biology laboratories, pharmacies and nurses performing SARS-CoV-2 tests. As with the cholera alerts in Haiti (18), these COVID-19 hotspots proved to be a valuable synthetic indicator to guide response interventions and to assess their targeting relevance. We therefore believe that such a mapping tool should have been used in other regions of France, and could be adapted to other contexts and future public health events.

As a matter of fact, the higher the hotspot level in the previous 3 weeks, the more the CORHESAN team responded with testing interventions. The team also prioritised areas of socio-economic deprivation and/or under-testing, and areas with high COVID-19 hotspot levels. But CORHESAN also seemed to concentrate the interventions in areas close to its Hôpital Européen headquarters, not only because this was where most hotspots and deprived and under-tested populations were concentrated, but perhaps also because organising community or door-to-door testing sessions in wealthier single-family residential areas in the South of the city proved more difficult and was perceived as less useful by the team. Finally, response to hotspots seemed less likely during weeks with more active health mediators, probably because the number of hotspots exceeded response capacity during the main epidemic waves (data not shown). Unfortunately, the targeting of vaccination outreach activities could not be evaluated because vaccination coverage was not available at the fine geographical scale in France. The fact that several hundred first doses were still being administered in 2022, may indicate that the CORHESAN’s outreach activities likely helped to motivate individuals long after the government introduced the sanitary pass in July 2021.

The intervention’s non-experimental nature and the intricate dynamics of the pandemic made it impossible to assess the impact of CORHESAN’s outreach interventions in the response to the COVID-19 pandemic in Marseille. But in the USA, culturally tailored outreach intervention with community health promoters and community-based walk-up sites proved effective to increase COVID-19 testing in vulnerable and hard-to-reach populations in Oregon (44) and New Orleans (61), respectively.

In conclusion, the CORHESAN intervention demonstrated the potential for innovation to address complex and dynamic public health challenges. This was a spin-off of the public health crisis, which stimulated creativity, streamlined administrative procedures, provided exceptional funding for pandemic response activities, and promoted collaborative partnerships, particularly between health authorities, researchers and field actors, and between health and social services sectors. This present evaluation thus provides important lessons on how outreach interventions with health mediation can be designed, implemented and evaluated. Such a strategy should be considered systematically when managing public health crises. When the COVID-19 crisis ended in June 2022, this experience paved the way for a new health mediation and outreach initiative in Marseille, which aims at promoting cancer screening and vaccination catch-up in deprived neighbourhoods, and is implemented by CORHESAN in the 1st, 2nd and 3rd central districts and by the SEPT association in the 13th, 14th, 15th and 16th northern districts. This time, the interventions are accompanied by an ambitious and multimodal prospective evaluation strategy, in order to provide convincing evidence and, hopefully, establish health mediation as a legitimate pillar of the French health system to address social and territorial health inequalities. A recent government report emphasises the need to improve access to training in health mediation. It advocates for the formal recognition of this profession, which would entail a training framework, inclusion in the national nomenclature of occupations and a salary scale. It strongly recommends evaluating the effectiveness and efficiency of health mediation and establishing relevant impact indicators (62). The present study on CORHESAN’s experience with COVID-19 and to the ongoing CORHESAN and SEPT cancer screening and vaccination project should therefore be seen as welcome pioneering efforts.

## Data availability statement

The anonymised raw data supporting the conclusions of this article will be made available by the authors upon reasonable request and the signing of a material transfer agreement. All data were routinely collected as part of the activities of this hospital-based outreach and prevention intervention, in accordance with the European GDPR (General Data Protection Regulation). Present implementation analyses were retrospectively conducted with authorisation #2023_17_10_SR of the ethical committee of the Hôpital Européen Marseille. All data were anonymised before analysis.

## Ethics statement

The studies involving humans were approved by Ethical committee of the Hôpital Européen Marseille (authorisation #2023_17_10_SR). The studies were conducted in accordance with the local legislation and institutional requirements. Written informed consent for participation was not required from the participants or the participants’ legal guardians/next of kin in accordance with the national legislation and institutional requirements.

## Author contributions

AF: Conceptualization, Data curation, Investigation, Methodology, Resources, Validation, Writing – original draft, Writing – review & editing. JG: Conceptualization, Funding acquisition, Methodology, Supervision, Validation, Writing – review & editing. FF: Conceptualization, Data curation, Formal analysis, Funding acquisition, Investigation, Methodology, Visualization, Writing – review & editing, Resources, Validation. SN: Conceptualization, Data curation, Formal analysis, Investigation, Methodology, Visualization, Writing – review & editing, Resources, Validation. AD: Data curation, Funding acquisition, Project administration, Resources, Supervision, Writing – review & editing. EL: Conceptualization, Formal analysis, Writing – review & editing. DB: Funding acquisition, Project administration, Resources, Supervision, Writing – review & editing. ML: Funding acquisition, Project administration, Writing – review & editing. LT: Writing – review & editing. PM: Funding acquisition, Writing – review & editing, Conceptualization, Investigation, Methodology, Project administration, Supervision, Validation. PC: Conceptualization, Funding acquisition, Project administration, Supervision, Writing – review & editing, Investigation, Methodology. SR: Project administration, Supervision, Writing – review & editing, Investigation, Methodology, Data curation, Formal analysis, Validation, Visualization, Writing – original draft, Conceptualization, Funding acquisition.
